# Explaining the adoption and use of computed tomography and magnetic resonance image technologies in public hospitals

**DOI:** 10.1186/s12913-021-07225-2

**Published:** 2021-11-27

**Authors:** Francisco Reyes-Santias, Manel Antelo

**Affiliations:** 1 GEN Universidade de Vigo, Vigo, Spain; 2 Universidade de Santiago de Compostela, Santiago de Compostela, Spain

**Keywords:** CT adoption, MRI adoption, CT use, MRI use

## Abstract

**Objective:**

This article examines what the adoption and use of advanced medical technologies – computed tomography (CT) and magnetic resonance imaging (MRI) – by public hospitals depend on and to what extent.

**Methods:**

From a sample of panel data for all public hospitals in the health service of Galicia (a subregion of the Galicia-North of Portugal Euroregion) for the 2010–2017 period, we grouped explanatory variables into inputs (resources), outputs (activities) and socio-demographic variables. Factor analysis was used to reduce as much as possible the number of analysed variables, discriminant analysis to examine the technologies adoption decision, and multiple regression analysis to investigate their use.

**Results:**

Factor analysis identified motivators on adoption and use of CT and MRI medical technologies as follows: hospital inputs/outputs (Factor 1); radiology studies and adoption of CT by public hospitals (Factor 2); research/teaching role and big-ticket diagnostic and therapeutic (lithotripsy) technologies (Factor 3); number of transplants (Factor 4); cancer diagnosis/treatment (Factor 5); and catchment area geographical dispersion (Factor 6). Cronbach’s alpha of 0.881 indicated an acceptable degree of reliability of the factor variables. Regarding adoption of these technologies, Factor 1 is the most influential, explaining 37% of the variance and showing adequate global internal consistency, whereas Factor 2 is limited to 13% of the variance. In the discriminant analysis, values for Box’s M test and canonical correlations such as Wilks’s lambda for the two technologies underpin the reliability and predictive capacity of the discriminant equations. Finally, and according to the regression analysis, the factor with the greatest influence on CT and MRI use is Factor 2, followed by Factors 1 and 3 in the case of CT use, and Factors 3 and 5 in the case of MRI use.

**Conclusion:**

CT and MRI adoption by public hospitals is mainly determined by hospital inputs and outputs. However, the use of both medical technologies is mainly influenced by conventional radiology studies and CT adoption. These results suggest that both choices – adoption and use of advanced medical technology – may be separate decisions as they are taken possibly by different people (the former by managers and policymakers and the latter by physicians).

## Introduction

A significant percentage of increased hospital expenditure comes from the use of more inputs per unit of final output. The input that has experienced greatest growth in recent decades has been medical technologies [1], accounting for 33–50% of increased healthcare spending [2]. While the adoption and use of new medical technologies improves the quality of medical care by improving health outcomes, those technologies contribute to the continuing rise in healthcare expenditure [3]. Consequently, a crucial issue in ultimately determining healthcare policies in most OECD countries is the development and adoption of innovative medical technologies in a context of exacerbated healthcare spending resulting from major demographic changes linked to greater life expectancy and ageing. According to Greenberg and Pliskin [4], technology adoption decisions pose a particular challenge to decisionmakers, as timely decisions regarding new technologies are often required before there is definitive evidence on clinical efficacy and economic merit.

Budget constraints for public hospitals and expected profits for private hospitals should not, however, be the only criteria determining technology adoption in hospitals. Puig i Junoy [5] suggest that innovation adoption in hospitals depends on the characteristics of the technology itself (i.e. the marginal advantage over the previously used technology), the objectives of the hospital as a firm (i.e. the characteristics of the entity adopting the technology), and the characteristics of the market.

The literature on health economics confirms that computed tomography (CT) and magnetic resonance imaging (MRI) are key healthcare technologies. For instance, Espargallés et al. [6] list CT and MRI among the 30 technologies receiving the most general medicine and primary care bibliometric citations in the last 25 years, while Collado et al. [7] and Robin et al. [8] highlight clear CT and MRI advantages over conventional radiology, as do protocol guides on appropriate diagnostic technologies issued by the American College of Radiology [9], the European Commission [10] and the Royal College of Radiologists [11].

While the adoption and use of CT and MRI technologies in place of more invasive and higher-risk approaches is gaining momentum, given their importance in diagnosing severe injuries, in the interest of monitoring and controlling health spending the high cost of acquiring and operating this equipment makes their evaluation necessary [12, 13]. Thus, exploring the factors that may explain CT and MRI adoption (the number of units acquired) and use (the number of scans performed) could shed light on these key healthcare innovations that have boosted growth in health expenditure in recent decades. This is the goal of this paper: to identify and evaluate the role played by public hospital inputs and output as well as socio-demographic characteristics of catchment areas in the adoption and use of new diagnostic and therapeutic procedures according to real healthcare needs.

This issue has been explored in the literature from several perspectives. Recently, Sandoval et al. [14] examined the relationship between hospital adoption and use of CT scanners and MRI machines and inpatient mortality and length of stay. They particularly investigated the role of adoption and use of medical imaging within a broader framework of hospital care where adoption is proposed to be a central, linking factor between hospital structural characteristics, market factors and hospital outcomes. Likewise, He et al. [15] found that population, GDP, and the number of hospitals, health professionals, hospital beds, outpatient visits, and inpatient visits all had a positive correlation with the allocation number of CT and MRI scanners, and, particularly, that the number of health professionals and the number of beds had a much closer correlation than other variables.^1^ Furthermore, in assessing the association of hospital characteristics and diagnosis with repeated utilization of CT and MRI, Chen et al. [18] pointed out that repeat use of CT and MRI within 90 days is high and is related to both diagnosis and hospital characteristics.

The remainder of the article is organized in four sections. First, the material and methods used in the research is outlined. Next, we offer the results obtained, followed by a discussion of those results. The paper concludes with some final remarks.

## Material and methods

The research scope covers public hospitals with CT and MRI technologies in Galicia (north-west Spain) – part of the Galicia-North Portugal Euroregion – in the period 2010–2017 (the most recent data available from the Ministry of Health in Spain). The sample includes all 14 public hospitals (whose capacity amounts to 7599 beds) that provide healthcare services to the Galician Health Service (SERGAS), employing 4651 medical specialists, equipped with 33 CT units and 13 MRI units, and implementing 290,080 CT scans and 117,372 MRI scans over this period.

Data on hospital inputs and outputs and on catchment population socio-demographic characteristics were sourced from hospital catalogues, system-wide hospital reports published by SERGAS, and data and statistics on specialized health centres and inpatient health establishments published by the Spanish Ministry of Health. All these data are publicly accessible to anyone interested in them.^2^

Measured as yearly hospital inputs were the following: number of beds; number of doctors, medical specialists, surgical specialists, radiologists and resident interns; and number of CT, MRI, haemodynamic, gamma camera, lithotripsy and linear accelerator units. On the other hand, hospital inputs and hospital outputs were measured as average diagnosis-related group (DRG) weight expressed as the case-mix index (CMI), average stay^3^ and number of consultations, admissions, emergencies, emergency admissions, surgical interventions, conventional radiology studies,^4^ CT and MRI scans and kidney/heart/liver transplants. Finally, catchment area socio-demographic characteristics were defined as inhabitants, population density and population aged above 65 years.

To determine the smallest possible number of components that would explain most of the observed variation we used factor analysis; specifically, the correlation matrix as the procedure [21] and orthogonal varimax as the rotation method [22]. On the other hand, we used discriminant analysis for predictive purposes, i.e. to determine whether or not a public hospital would acquire a CT or MRI unit. Belongingness of studied elements to one or another group was introduced into the analysis through a qualitative variable that took as many values ​​as the number of groups. This variable played the role of the dependent variable, while the independent variables were considered to be discriminant variables. The two dependent variables are the number of public CT units and the number of public MRI units. Both were considered as dichotomous categorical variables, whose values ​​were recorded to values ​​0 and 1, indicating the absence and the presence of the quality corresponding to one of the two categories, respectively. On the other hand, the selected independent variables were the factors obtained in the factor analysis.^5^ Finally, multiple linear regression was used to predict CT and MRI use, i.e. the number of scans performed with those technologies [5]. The sample data used to perform the regression analyses were panel data and the estimation method used was ordinary least squares; significance was established at *p* < 0.05.

## Results

To explain the maximum percentage of variance and an acceptable parsimony of the exploratory factor analysis, we set the percentage of explained variance to 86%, which is verified for the six factors detailed below (expressed as numbers except where otherwise indicated):

- **Factor 1 (hospital inputs and outputs):** beds, surgical specialists, medical specialists, radiology specialists, emergency admissions, emergencies, admissions, surgeries, consultations, and average stay (days).
- **Factor 2 (conventional radiology studies and adoption of CT by public hospitals):** conventional radiology studies, and public CT units.
- **Factor 3 (research/teaching role and big-ticket diagnostic (MRI) and therapeutic (lithotripsy) technologies):** resident interns, Spanish Health Research Fund (FIS) grants, lithotripsies, and public MRI units.
- **Factor 4 (transplants):** heart/kidney/liver transplants.
- **Factor 5 (cancer diagnosis/treatment):** linear accelerator units and gamma cameras, and CMI.
- **Factor 6 (catchment area geographical dispersion):** population density measured as inhabitants/km^2^.

Table 1 below summarizes the results provided by the factor analysis and the percentage of variance explained by each factor after axis rotation.

**Table 1:** Factor analysis and total explained variance

	Factor	1	2	3	4	5	6
**Initial eigenvalues**	Total	17.941	2.539	1.948	1.684	1.498	1.07
	% variance	57.873	8.189	6.284	5.432	4.831	3.45
	% accumulated	57.873	66.062	72.346	77.778	82.609	86.059
**Extraction sums of squared loadings**	Total	17.941	2.539	1.948	1.684	1.498	1.070
	% variance	57.873	8.189	6.284	5.432	4.831	3.450
	% accumulated	57.873	66.062	72.346	77.778	82.609	86.059
**Rotation sums of squared loadings**	Total	11.601	4.046	3.402	3.303	3.123	1.203
	% variance	37.422	13.053	10.974	10.655	10.076	3.880
	% accumulated	37.422	50.475	61.449	72.103	82.179	86.059
**Cronbach’s alpha**	0.881	0.880	0.860	0.797	0.868	0.886	0.850

As can be seen in Table 1, Factor 1 (hospital inputs and outputs) explains by far the highest percentage of variance after rotation, while percentages explained by Factors 2, 3, 4 and 5 are similar, but much lower. Finally, while Factor 6 (catchment area geographical dispersion) only explains 3% of the variance, the variable is, a priori, important for Galicia because is a region characterized by a highly dispersed population.

The value of the Kaiser–Meyer–Olkin (KMO) test we obtained was 0.878, indicating that the sample can be considered suitable for factor analysis [22]. On the other hand, Bartlett’s sphericity test resulted in significance lower than the *p*-value of 0.05, so the hypothesis that the variables were uncorrelated in the population (null hypothesis) was rejected. To ensure both an explanation of maximum variance in the variables and an acceptable model parsimony resulted, we set the percentage of explained variance to 86%. Finally, the Cronbach’s alpha value of 0.881 reflects an acceptable degree of reliability. Then, we rotated the factors in a multidimensional space [27] using the varimax orthogonal rotation criterion [22].

As the orthogonal rotation method (which rotates axes orthogonally at the same angle) we used the varimax method, which tries to minimize the number of variables with high saturations by the same factor. The importance of each factor was evaluated considering the proportion of variance explained by the factor after the rotation. The results of the rotated factorial solution with the varimax method are summarized in Table 2.

**Table 2:** CT and MRI adoption: discriminant analysis results

Factor	Adoption of CT units	Adoption of MRI units
Factor 1	0.935	1.255
Factor 2	−0.243	0.722
Factor 3	0.170	0.195
Factor 4	0.437	0.314
Factor 5	0.396	−0.538
Factor 6	0.272	0.334
Box’s M test	215.77	307.89
*p*-value	0.003	0.000
Wilks’ lambda	0.710	0.373
Canonical correlation	0.538	0.792
Correctly classified	80.6%	93.5%

Table 2 shows the output of the discriminant equations regarding the adoption of CT and MRI technologies. In both cases, the corresponding Box’s M test and the degree of significance of the *p*-value indicate that the variance-covariance matrices for each group or level of the variable differ significantly. In turn, the canonical correlations of 0.538 for CT adoption and of 0.792 for MRI adoption show good discrimination of the function. Finally, the Wilks’ lambda of 0.710 with a *p*-value of 0.003 confirms the significance of the discriminant function and its explanatory capacity. Also, for MRI adoption, the Wilks’ lambda of 0.373 with a p-value of 0.000 corroborates its explanatory capacity, as the variables take significant values, given the coefficients of determination. Both facts suggest that the factors can be potentially explanatory.

According to Table 2, Factor 1 (hospital inputs and outputs) most affects CT adoption, while Factor 3 (research/teaching role and big-ticket diagnostic (MRI) and therapeutic (lithotripsy) technologies) has very little influence on CT adoption; this would suggest that adoption of CT technology has plateaued. Finally, Factor 2 (conventional radiology studies and adoption of CT by public hospitals) impacts negatively on CT adoption, which would suggest that CT could be a substitute technology of conventional radiology. This, in turn, would tend to slow down CT adoption.

Regarding MRI adoption by public hospitals, three facts are of note. First, Factor 1 most impacts on adoption, even more so than for CT adoption. Second, and contrary to what happens with CT adoption, Factor 2 impacts positively on MRI adoption, which suggests that MRI scans complement conventional radiology studies. Third, and also contrary to what happens with CT adoption, Factor 5 (cancer diagnosis/treatment) has a negative impact on MRI adoption, probably due to greater MRI focus on functional studies or to substitution between MRI and nuclear medicine (gamma cameras) for tumour diagnostic purposes. Finally, the impact of population concentration on CT and MRI adoption by public hospitals is positive and slightly greater for MRI adoption than for CT adoption.

In Table 3 we summarize the results of the regression analysis regarding CT and MRI use.

**Table 3:** Estimates of CT and MRI use (number of diagnostic scans) with regression analysis of factors

	Number of CT scans	Number of MRI scans
Constant	3461.21 (0.001)	823.34 (0.067)
**Factor 1**	1945.80 (0.001)	426.110 (0.341)
**Factor 2**	−59,630 (0.242)	1857.25 (0.001)
**Factor 3**	1425.68 (0.006)	1304.20 (0.005)
**Factor 4**	1290.26 (0.013)	− 311.51 (0.486)
**Factor 5**	− 1255.21 (0.016)	578.840 (0.198)
**Factor 6**	− 457.44 (0.162)	75.45 (0.866)
Adj. R	0.787	0.675
Durbin–Watson test	0.808	1.897
F	52.090 (0.001)	29.73 (0.001)
Z (K-S)	1.254 (0.086)	1.163 (0.134)

**Note***:* Significance was established at *p* < 0.05

*P* values in parentheses.

According to Table 3, in public hospitals the factors with the greatest influence on CT use are Factor 2 (conventional radiology studies and adoption of CT by public hospitals), followed, in turn, by Factor 1 (hospital inputs and outputs) and Factor 5 (cancer diagnosis/treatment), all of which have positive signs, while the factors with the greatest influence on MRI use is Factor 2 followed by Factor 4 (transplants), both with positive signs. The results regarding Factor 6 (catchment area geographical dispersion) indicate that the more concentrated the population, the greater its influence on MRI use and the lower its influence on CT use.

For both CT and MRI use, the value for the F statistic and the associated *p*-value indicate significance for the model at 1%. In turn, the value of the Durbin–Watson statistic and the condition indices at less than 15 indicate that the model does not have autocorrelation or multicollinearity problems, respectively, while the Z value for the Kolmogorov–Smirnov test, with a *p*-value greater than 0.05, indicates that the residuals of the equation are normally distributed.

The results obtained indicate that Factor 1 impacts positively and significantly on CT and MRI adoption by public hospitals. This factor allows 37.42% of the variance to be explained, shows adequate global internal consistency, and is more relevant to MRI than to CT adoption. However, Factor 1 is not the main determinant of CT and MRI use: the main determinants of CT use are conventional radiology studies and adoption of CT by public hospitals, whereas the main determinants of MRI use are the hospital research/teaching role and big-ticket diagnostic (MRI) and therapeutic (lithotripsy) technologies. The fact that population density plays a statistically significant role in the demand for MRI but not in the demand for CT suggests that there is demand induction for CT, but not for MRI. Finally, the impact of the case-mix index (CMI) is reflected in positive and significant correlations for use (the number of scans) for CT with transplants, and for MRI with cancer diagnosis and treatment.

The fact that Factor 1 (hospital inputs and outputs) is that which most influences the CT and MRI adoption decision, whereas Factor 2 (conventional radiology studies and adoption of CT by public hospitals) is that which most influences the actual usage of those technologies allows us to infer that choices as to adoption and use of advanced medical technologies may be separate decisions, a consequence, perhaps, of different decision-making processes. This suggests a possible dissonance between decisions on adoption and decisions on use, indicating, in turn, the need to guide decision-makers in better planning investments in medical technologies so as to avoid premature diffusion without adequate knowledge of true effectiveness.

## Discussion

Our findings regarding the significantly positive correlation between hospital inputs and outputs and the number of CT and MRI units would suggest that the more units are installed, the greater the tendency of doctors to use them [28]. In line with this, Harstall [29] reported that hospital size, teaching role, greater specialization, research activity and resource availability were positively associated with technology adoption. In turn, Abedini et al. [30] concluded that the main factors influencing CT and MRI adoption were the number of beds, doctors and patients and hospital location, whereas Hong [31] showed that the number of CT units reflected bed and specialty numbers and, most especially, location in larger cities. Finally, Hall [32] found that greater use of diagnostic technologies was associated with greater population density, more doctors per capita, a higher ratio of specialists to general practitioners and a higher percentage of doctors involved in teaching.

Among the characteristics and objectives of hospitals and their doctors is the extent to which innovation increases the prestige of the hospital. This is confirmed by the high coefficient observed for the transplant factor – an indicator of prestige and healthcare quality in a hospital. Our results are aligned with others [33–37], as well as Newhouse [38] regarding the quality of care. In the same vein, Dafny [39] has also shown that hospitals build a technological competitive advantage by experience acquired with medical technology. This behavior in the adoption of medical technology is rationalized by the fact that hospitals with MRI technology obtain an advantage over the other hospitals in the area, in terms of more referrals and expansion of the services they provide and also by the fact that technological level is an important issue in hospital choice by junior consultants [40]. As for the diffusion of medical technologies, Cromwell [34] and Greenhalgh et al. [41] show that this is slower in areas with fewer doctors per inhabitant, relating their finding to doctor pressures to introduce innovations.^6^

Our work also shows a positive coefficient sign for the research and teaching factor, i.e. the level and type of training offered also influences adoption decisions since, in general, medical staff are trained in hospitals equipped with the most sophisticated technologies [5], while Booth-Clibborn et al. [44] reported that research activities in a hospital have little influence on the adoption of MRI technology.

A possible explanation for the negative (statistically significant) coefficient for conventional radiology studies and CT adoption is that the adoption rate of a new technology will be reduced if the innovation replaces very durable equipment [45], as the outdated technology would still hold a relatively long service life.^7^ The resistance to abandon obsolete technologies can also be explained as a natural defence of past investments in time, effort and money made in incorporating and learning technologies; in many cases, the same agents that plead for an innovation are those that resist abandoning older technologies [46]. On the other hand, the coefficient for radiology and MRI adoption could suggest that MRI complements rather than substitutes for other radiological techniques, as reported by Capdevila [47] in establishing the complementarity of MRI with other diagnostic techniques (particularly, CT), by Lenz et al. [48] in explaining that MRI allows new or safer interventional procedures to be performed, and also by Ackerman et al. [49] and Vita et al. [50], who established that MRI is a complementary technology rather than a substitute for other diagnostic imaging technologies.

The negative sign of the cancer diagnosis and treatment factor coefficient relative to MRI adoption seems to be explained by the substitution between MRI and nuclear medicine technologies. This hypothesis is endorsed by the guidelines and protocols of the American College of Radiology [9] and the European Commission [10].

The positive and significant coefficient sign for hospital inputs and outputs in explaining CT use (the number of scans performed with this technology) are aligned with Caicoya et al. [51]‘s results. In fact, in studying CT and MRI use, these authors show that the higher rate of CT and MRI use is associated with a higher supply of CT and MRI equipment and a greater supply of specialists per hospital. Furthermore, Rodríguez-Álvarez and Lovell [52], in their research into excess capacity and spending behaviour in Spanish public hospitals, also found that hospital size and volume was directly associated with the number of X-ray studies performed.

We also found that the socio-demographic factor (population density) is non-significant in explaining CT use, whereas the opposite holds for MRI use. This finding allows us to infer, following Hay and Leahy [53] and Trost et al. [54], that there would be induced demand for CT scans, but not in the case of MRI scans.

Finally, the positive and significant coefficients for the transplant factor for CT use and for the cancer diagnosis and treatment factor for MRI use can be understood as a case-mix influence on demand, a finding supported by other studies of demand for CT and MRI explorations [55–58].

Summing up and based on our findings, we can frame our study within the need for health decision-makers to analyse and disseminate information on trends related to new health technologies in order to address potential deficits in their implantation, accessibility and use.

## Conclusions

The adoption and use of new medical technologies such as CT and MRI undoubtedly improves the quality of care for patients, but is also a main reason behind increased healthcare expenditure. The adoption and use of advanced technologies is thus permeated by a trade-off between improved care and increased spending, thereby posing a challenge for decision-makers, often faced with making decisions regarding these technologies in the absence of definitive evidence on their clinical efficacy.

To shed light on this issue, we explored CT and MRI adoption and use by public hospitals in Galicia (Spain), examining the main factors that influence the corresponding decisions. Our findings suggest that the factor that determines greater adoption of those technologies is hospital inputs and outputs, and that this factor is more important for CT adoption than for MRI adoption. However, this factor is not the main driver of actual use of those technologies (the number of scans). According to the multiple linear regression performed with the variable dependent on CT and MRI use, this is mainly explained by the radiology equipment and activity factor, which includes installed CT equipment. This factor has the greatest weight in the demand for CT and MRI studies – greater than the hospital inputs (number of beds, number of consultants, number of surgical theatres, etc.) and outputs factor (covering admissions, consultations, emergencies, surgical interventions, etc.). As for the coefficient reflecting the socio-demographic factor, this has no statistical significance, suggesting that there would be an induction of demand for CT technology use.

The insights provided by this study into the factors that underlie the adoption and use of new medical technologies can help technology decision-makers in public hospitals. Detailed assessments of the adoption and predicted use of medical technologies play a crucial role in avoiding premature diffusion of technology prior to accurate evidence of its effectiveness. Our study can also contribute to predicting the effects of publicly funded programs of assistance to certain disadvantaged population groups on technology dissemination processes.

Some limitations to our study can be traced. For example, although we have obviated the role of aspects such as policy factors (the electoral and political cycle), boom periods and bad periods or the introduction of public–private management experiences in the period considered, these aspects could undoubtedly affect the adoption and use of medical advanced technologies. However, the spirit of our paper is to analyse how the internal factors of the public health system could affect these decisions. The consideration of these and other aspects may allow a refinement of the approach and constitute a research avenue for the future.

## Abbreviations

CMI: Case-mix Index
CT: Computer tomography
DRG: Diagnosis-related group
FIS: Fondo Investigacion Sanitaria (Spanish Government Health Research Fund)
KMO: Kaiser–Meyer–Olkin test
MRI: Magnetic resonance imaging
SERGAS: Servizo Galego de Saúde (Galician Health Service)

## Footnotes

1 Even though Japan had fewer radiologists per capita than any other OECD country, in the past the CT installations grew three times faster in Japan than in the U.S. [16]. In that sense, Masatoshi et al. [17] showed that between 2006 and 2012 the number of radiologists increased by 21.7%, but the number of radiologists per 1000 CT (MRI) utilizations decreased by 17.9% (1.0%).

2 The data of the SERGAS reports and the set of public hospitals are available at https://www.sergas.es/A-nosa-organizacion/Publicaci%C3%B3ns-da-Organizaci%C3%B3n?idioma=es. In turn, the data with information provided by the Spanish Ministry of Health are available at https://www.mscbs.gob.es/estadEstudios/estadisticas/estHospiInternado/inforAnual/homeESCRI.htm.

3 We follow Ellis and McGuire [19] in considering that average stay is a suitable approach to evaluating health resource use.

4 This quantitative variable refers to the total number of diagnostic imaging studies with conventional radiological techniques (general radiography, mammography, ultrasound, radiography and digital angiography), whether simple or dynamic, with or without contrast, performed on a patient in one session and for a specific purpose [20].

5 The choice of methodology would be between discriminant analysis and logit or probit, but discriminant analysis was chosen because it has a greater predictive capacity than the other two, as pointed out by a number of authors [23–26].

6 However, authors such as Rapoport [42] and Willems [43] have pointed out in their studies on the adoption of CT technology that there is no relationship with the number of doctors per inhabitant. Their argument is that when there are few doctors per inhabitant, hospitals compete in the adoption of technology to attract medical personnel.

7 However, in their study, this variable of obsolete equipment duration was not statistically significant.
